# Type 1 diabetes and hyperthyroidism in a family with celiac disease after exposure to gluten: a rare case report

**DOI:** 10.1186/s40842-018-0075-2

**Published:** 2018-12-19

**Authors:** Azita Ganji, Meysam Moghbeli

**Affiliations:** 1 Department of Gastroenterology and Hepatology, Faculty of Medicine, Mashhad University of Medical Sciences, Mashhad, Iran; 2 Gastroenterology and Hepatology Research Center, Mashhad University of Medical Sciences, Mashhad, Iran; 3 Department of Modern Sciences and Technologies, Faculty of Medicine, Mashhad University of Medical Sciences, Mashhad, Iran

**Keywords:** Celiac disease, Type 1 diabetes, Hyperthyroidism

## Abstract

**Background:**

Celiac disease (CD) is an autoimmune disorder related to the gluten and can be also associated with some other endocrine disorders such as type 1 diabetes and thyroid disease. Gluten exposure in CD may have especial role in developing other auto immune disorder.

**Case presentation:**

We reported two familial cases with celiac disease who were on a gluten free diet (GFD) and hyperthyroidism and type 1 diabetes were appeared following a regular diet. Their autoimmune disorders were ameliorated after avoidance of dietary gluten.

**Conclusions:**

These cases highlighted the role of gluten exposure in developing other autoimmune disorders associated with CD, especially in young patients whom they are not cooperative to keep GFD. We recommended to evaluate the organ specific antibodies for risk assessment in these patients.

## Background

Celiac disease (CD) is an autoimmune enteropathy triggered by gluten which is a storage protein in various cereals. CD has been reported to be associated with some other endocrine disorders such as type 1 diabetes (T1D) and thyroid disease. The incidence of T1D and thyroid disease is 5% among CD cases. The higher prevalence of additional auto immune disorders has been shown in CD who were on GFD with OR > 3 compared with controls [1]. Although, gluten is the main cause of CD, it has also an important role in development of other auto immune disorder [2]. Duration of gluten exposure is an important factor for the progression of autoimmune disorders. Early onset of CD and absence of gastrointestinal symptoms have been also shown as risk factors for the other autoimmune disorder [3]. CD and T1D are complex disorders with shared genetic components. HLA-DQ2 haplotype is observed in about 90% of CD cases and in more than 50% of T1D patients. While, HLA-DQ8 has been observed in about 10% of CD patients and about 70% of T1D cases [4]. Role of gluten consumption, gut permeability, and inflammation are also reported in T1D [5]. On the other hand, autoimmunity related tissue damage and intolerance to dietary antigens may be a feature of T1D [6]. Gluten affects the tight junction of epithelial cells and facilitates entrance of antigens from lumen and activation of immune cells in epithelial cells [7, 8]. In another study they showed gluten can induce and increase in gut permeability even in non-celiac individuals [9]. High prevalence of auto immune disorder in CD can be explained by common genetics and environmental factors. In addition, it has also been demonstrated that the anti TTG IgA antibodies react with thyroid tissue, and this binding can be contributed to the thyroid disease in CD cases. Leaky gut causes alterations in the systemic inflammatory responses and autoimmunity due to gluten intake and leads to affect remote organs including the thyroid. Predispose to autoimmune diseases by intestinal permeability has been reported in CD [10, 11] and thyroid dysfunction [12]. There is a relationship between gut and thyroid [12, 13]. Moreover, the anti-t TG titers are correlated with TPO antibody titers [13]. Increased prevalence of CD-associated antibodies has been also shown in autoimmune thyroid disease [14]. Therefore, celiac-associated autoantibodies are contributed to the thyroid dysfunction in CD.

## Case presentation

### Case 1

A 16 years old male, who was diagnosed as celiac disease by family screening. Anti TTG levels were 89 Ru/ml (normal: up to 20) with normal total IgA (without any gastrointestinal symptom). Upper endoscopy was done and nodularity in bulb and second part of duodenum was observed (biopsy was obtained). Pathological examination showed marsh 3a. Laboratory tests also showed low levels of 1, 25(OH) D3 < 8, Hb: 12.4 g/dL, AST: 31 U/L, ALT: 15 U/L, ALP: 905 U/L, TSH: 1.9 mIU/L, Anti TPO: 24 IU/Ml, calcium: 10 mg/dl, and phosphore: 6.8. In his genetic study DQ2 was positive and DQ8 was negative. His TTG levels approached to the near normal levels of TTG (27 Ru/ml) after 2 years of gluten free diet (GFD). He started to have a regular diet arbitrarily and anti TTG levels got back to 110 Ru/ml without any symptom of gastrointestinal problem. After one more year of regular diet, he referred with chief complains of weight loss (about 10 kg), polyuria, and polydipsia. Blood sugar was 570 and he admitted to the hospital with an insulin therapy. He started the GFD and regular and long acting insulin to decrease blood sugar. He had no familial history of diabetes. After about 3 months, he had episode of hypoglycemia and we started dose reduction of insulin in 1 month. Finally, his FBS got back to the normal level by low dose of insulin (anti TTG level was 56 Ru/ml). Two months after GFD, we stopping insulin and after 2 months of follow up he still had normal FBS: 99 and HbA1C was 7%, but level of anti-islet cell was 7.3 IU/ml and glutamic acid decarboxylase was 200 IU/ml. He started to have gluten few times a week after about 4 months and TTG raised, and again he got symptomatic T1D. Now he is on insulin for control of FBS even with strict GFD and his Hb A1C is 7.7.

### Case 2

Sister of first case who was 14 years old female, had been referred with chief complains of dyspepsia, anemia, and oral aphtha. Level of anti TTG was 274 RU/ml with a normal total IgA. Endoscopy with duodenal biopsy was also done for this case and there was scalloping and fissuring in bulb and second part of duodenum. Duodenal biopsy was obtained and pathological tests showed marsh 3c. Other laboratory tests also showed Hg: 8.8, SGOT: 18 U/L, SGPT: 15 U/L, ALP: 308 u/l, 1, 25(OH) D3: 4 ng/mL, Ca: 9.8 mg/dl, TPO: 69 IU/ml, and normal TSH level. DQ2 was positive and DQ8 was negative. She had low levels of selenium (80 μg/L) and zinc was normal (899 μg/L). She started a gluten free diet and levels of TTG dropped to 50 Ru/ml. HCT was 35.7% and her symptoms were getting much better without any abdominal pain. After 2 years, she started on regular diet arbitrarily and after 1 year she referred to the clinician by chief complains of weight loss and tremor. In her laboratory tests, anti TTG level was more than 200 Ru/ml, TSH < 0.005, T3: 360 ng/ml, T4: 16.4 mμg/dl. After 4 months of GFD her symptoms got better and TSH get back to normal and methymazol was tapered without any problem. After 1 year she is in GFD and low level of anti TTG and she is still on remission of hyperthyroidism without any treatment.

## Discussion

Continued gluten exposure in untreated CD might lead to the other autoimmune disorders in cases who have a susceptible genetic [7]. GFD can reverse the autoimmune process and prevent its severity and sometimes life-threatening complications. Gluten free diet in CD with high risk of autoimmune disease can protect them against the Insulin dependent diabetes and hyperthyroidism. Leaky gut in untreated CD makes them predispose to different antigen originated from microbiota and foods in small bowel. It can pass over tight junction and present to immune system and trigger a systemic autoimmune reaction. Our hypotheses are to measure serum level of anti-islet cell antibody and glutamic acid decarboxylase to predict the possibility of Insulin dependent diabetes in young CD cases, which may have gluten exposure or who are not cooperative to keep GFD. Fuchenbusch et al. showed a GFD may lack any protective effect in cases with a positive family history of T1D with islet-autoimmunity [15]. However, in another study, immune response to the islet antigens in celiac cases disappeared following a GFD [16]. Most reported investigations in this area involve the animal model studies. However, in a case report, remission was achieved in a male child with insulin-dependent diabetes without insulin therapy on a GFD [17]. GFD in patients with high anti TPO may also protect against overt autoimmune thyroid disease. Although, the prevalence of hypothyroidism among CD patients was significantly increased, hyperthyroidism was not significantly different in comparison with control groups [18]. Organ –specific autoantibodies can have a predictive role in development of other autoimmune disorders associated with CD but we do not know yet, how necessary is to measure islet cell antibody level and anti thyroperoxidase in celiac patients who are not cooperative about keeping GFD.

In conclusion, Gluten exposure can induce overt and life threatening autoimmune disorder in CD with inducing organ –specific antibodies. We should consider the possibility of developing other autoimmune disorders in CD cases who are not on GFD, and ameliorating with GFD, especially in young CD patients .

## Abbreviations

CD: Celiac disease
GFD: Gluten free diet
T1D: Type 1 diabetes
